# Efficacy of Extracorporeal Shockwave Therapy on Calcified and Noncalcified Shoulder Tendinosis: A Propensity Score Matched Analysis

**DOI:** 10.1155/2019/2958251

**Published:** 2019-03-14

**Authors:** Kuan-Ting Wu, Wen-Yi Chou, Ching-Jen Wang, Chen-Yu Chen, Jih-Yang Ko, Po-Cheng Chen, Jai-Hong Cheng, Ya-Ju Yang

**Affiliations:** ^1^Department of Orthopedic Surgery, Kaohsiung Chang Gung Memorial Hospital, Graduate Institute of Clinical Medical Science, Chang Gung University College of Medicine, Kaohsiung, Taiwan; ^2^Center for Shockwave Medicine and Tissue Engineering, Department of Medical Research, Kaohsiung Chang Gung Memorial Hospital, Graduate Institute of Clinical Medical Science, Chang Gung University College of Medicine, Kaohsiung, Taiwan; ^3^Department of Physical Medicine and Rehabilitation, Kaohsiung Chang Gung Memorial Hospital, Graduate Institute of Clinical Medical Science, Chang Gung University College of Medicine, Kaohsiung, Taiwan

## Abstract

**Background:**

Extracorporeal shock wave therapy (ESWT) had been proved to be beneficial in calcific tendinosis; however, the treatment efficacy in noncalcific tendinosis of rotator cuff still remains controversial. The present study was to compare the outcomes ESWT among the noncalcific rotator cuff tendinosis and different types of calcific tendinosis on the basis of similar shoulder functional status.

**Methods:**

A retrospective, comparative study was conducted with the enrollment of 20 patients in each group through propensity score matching in a 1:1:1 ratio from 291 patients who underwent single ESWT for painful shoulder tendinosis. The patients were divided into three groups which included noncalcified tendinosis (NCTS), type I dense calcified tendinosis of shoulder (DCTS), and type II and type III translucent calcified tendinosis of shoulder (TCTS) according to Gartner and Heyer classification. The clinical evaluation included the subjective pain score with visual analog scale (VAS) and functional outcome with Constant and Murley score (CMS).

**Results:**

Twelve months after ESWT, the VAS in TCTS (1.5 ± 2.48) was statistically significant lower than NCTS (2.9 ± 2.86) and DCTS (3.8 ± 2.46) (p=.011). For the functional outcome, the overall CMS was superior in TCTS than the NCTS and DCTS (86.9 ± 19.7 versus 78.7 ± 18.3 and 71.1 ± 17.8, p=.014). Besides, the subscales of pain score, strength, and range of motion in TCTS improved significantly better than NCTS and DCTS. 70% of patients were complaint-free in TCTS group, which was higher than the NCTS group (15%) and DCTS group (25%) (p<0.05).

**Conclusion:**

The present study indicated that the high-dose ESWT posed superior clinical efficacy in type II/III calcification tendinosis rather than type I calcification and noncalcific shoulder tendinosis.

## 1. Introduction

The prevalence of shoulder complaints ranges from 16 to 34% in the general population and increases with advancing age [1, 2]. According to the literature, the incidence of rotator cuff disease is 9.7% in patients younger than 20 years and 62% in patients older than 80 years of age [3]. In addition, the estimated incidence of rotator cuff tendinosis ranges from 0.3 to 5.5% [4]. Although several pathomechanisms have been proposed for rotator cuff tendinitis, they remain controversial. The intrinsic and extrinsic mechanisms are commonly accepted as referring to vascular and biomechanical factors. The intrinsic mechanism presumes that compromise of the microcirculation system of the rotator cuff tendon results from overload and degeneration, which further impedes tissue-healing. On the other hand, the extrinsic mechanism implies that subacromial impingement by surrounding structures, such as the acromion, coracoacromial ligament, acromioclavicular joint, and coracoid process, results in repetitive injury of the rotator cuff tendon [5, 6].

Although rotator cuff tendinitis is usually self-limiting in the clinical setting, chronic symptomatic tendinosis occurs in some particular patients. Conservative treatment has been regarded as the first-line therapy for rotator cuff tendinitis, including rest, nonsteroidal anti-inflammatory drugs (NSAIDs), physiotherapy, corticosteroid injection, or dry needling. However, the effectiveness of these treatments is still not well-established [7, 8]. Extracorporeal shockwave therapy (ESWT) appears to be a promising alternative and has been proven to be beneficial in several musculoskeletal diseases and especially enthesopathies [7, 9], including plantar fasciitis [10], elbow epicondylitis [11], patella tendinitis [12], and Achilles tendinitis [13]. Although the exact treatment mechanism of ESWT for chronic tendinosis has not been determined, it has been suggested that ESWT induces early release of angiogenic and proliferating growth factors, with a positive effect on neovascularization of the tendon, which may reactivate the regeneration potential [14]. These findings implied that tissue regeneration therapy, like ESWT, has a positive influence on the healing of chronic tendinosis, which is characterized by insufficient inflammatory cells and hypovascularity. For the treatment of rotator cuff tendinosis, ESWT has proved effective in functional improvement and pain reduction of calcific tendinitis [15]. However, further investigation demonstrated that Gardner type I calcification is the major negative prognostic factor [16, 17]. In addition, recent evidence has presented inconsistent results for ESWT in the treatment of noncalcific tendinosis of the shoulder [18, 19].

According to a review of the literature, no comparative study regarding the efficacy of ESWT in calcific tendinosis and noncalcific tendinosis has been performed. Therefore, we conducted a match-controlled study to demonstrate the comparative outcomes of ESWT for the treatment of noncalcific rotator cuff tendinosis and different types of calcific tendinosis on the basis of a similar shoulder functional status.

## 2. Methods

### 2.1. Patients

This retrospective study was approved by our institutional review board (No. 201801188B0). Patients with symptomatic calcific and noncalcific rotator cuff tendinosis treated with ESWT were systemically reviewed. The diagnosis was initially made by clinical symptoms, such as pain or disability, which lasted for more than six months, confirmed by ultrasonography or magnetic resonance image (MRI). The calcification identified as homogenous or heterogenous hyperdense areas of varying shape in the rotator cuff tendon. Patients who failed to respond to conservative treatment, such as physiotherapy, NSAIDs, or analgesics, for 3 months were referred for ESWT if they had no contraindications, which included pregnancy, coagulopathy, acute infection, or malignancy. Patients who had a full-thickness tear were also excluded from this analysis. To exclude the influence of external impingement by acromion morphology, the patients with type II or III acromion were not enrolled.

### 2.2. Treatment

All current treatments, including NSAIDs, aspirin, and physiotherapy, were discontinued two weeks prior to ESWT until four weeks after ESWT. The patients were placed in a supine or sitting position without anesthesia or analgesics. The electrohydraulic shockwave was produced using Ossastron (Sanuwave, Swanee, Georgia) or Orthospec equipment (Medispec Ltd., Yehud, Israel). 3000 impulses were delivered at 16 kv to 18 kv (0.32 mJ/mm^2^ energy flux density) with the Ossatron or at level seven (0.32 mJ/mm^2^) for the Orthospec under image guide on the tendon with calcific deposition and on the point of maximal tenderness in patients without calcification.

As Gartner type I calcification has been identified as one of the major negative prognostic factors for ESWT in calcific tendinosis, we categorized the patients into three groups: noncalcified tendinosis (NCTS), type I calcification (DCTS), and type II/III calcification (TCTS). According to the Gartner and Heyer classification system [20], type I calcification refers to well-circumscribed, dense, formative calcification, and type II calcification to clearly circumscribed, translucent, cloudy, and dense calcification. Type III calcification is cloudy, translucent and resorptive.

The age, gender, side of shoulder lesion, and duration of symptoms of the patients were recorded as the demographic data. Clinical evaluation included a subjective pain score using a visual analog scale (VAS) and the functional outcome according to the Constant-Murley score (CMS), which includes subscores of pain (0-10), night pain (0-5), strength (0-25), activities of daily living (0-20), and range of motion (0-40) [21]. The higher the score, the better the function, with range of 0 to 100. The parameters were recorded prior to ESWT and 3, 6, and 12 months after ESWT. A total of 291 patients with symptomatic shoulder tendinosis who underwent ESWT from 1998 to 2015 were retrospectively reviewed. Of the 291 eligible patients who met the inclusion criteria, 1:1:1 propensity score matching was performed, with potential confounders of functional outcome including age, gender, VAS, strength, activity, motion, and overall Constant score. The functional outcome at the 12-month follow-up point was adopted for comparison with baseline shoulder function and between three groups.

Regarding overall satisfaction, we defined patients with symptom improvement of more than 80% as complaint-free, 50-79% as significantly better, 25-49% as slightly better, and lower than 24% as unchanged. Patients who were considered “complaint-free” and “significantly better” at the final follow-up point were regarded as having a high level of satisfaction.

### 2.3. Statistical Analysis

The demographic data are presented as the mean ± standard deviation for continuous variables and a percentage for discrete variables with descriptive statistical analysis. Propensity score matching analysis was performed to control potential confounders. The Chi-square test was used to compare categorical variables, and the Wilcoxon signed-rank test and Kruskal-Wallis test were used for comparison of continuous variables between groups. Bonferroni correction was used for* post hoc* analysis after the Kruskal-Wallis test. A* p*-value lower than 0.05 was considered significant. All statistical analyses were performed using SPSS software V.21 (SPSS Inc. Chicago, IL, USA).

## 3. Results

### 3.1. Patient Demographic Characteristics

A total of 20 patients from each pool of patients were matched successfully. There were no significant differences with regard to age, gender, or side of the affected shoulder (Table 1). However, the duration of symptoms was longer in the DCTS group (20.3 ± 20.90 months,* p* = 0.016) than in the NCTS (13.4 ± 12.65 months) and TCTS groups (9.8 ± 9.3 months).

### 3.2. Clinical Results

There were no significant differences in the baseline pain score and shoulder function, including strength, activities of daily living, range of motion, and overall Constant score, among the three groups. One year after ESWT, VAS was decreased significantly in the NCTS (from 5.5 ± 0.76 to 2.9 ± 2.86, p= 0.001), TCTS (from 5.4 ± 1.04 to 1.5 ± 2.48, p<0.001), and DCTS groups (from 5.4 ± 0.94 to 3.8 ± 2.46, p= 0.02). Regarding functional outcome, the scores on the five subscales of the CMS and the overall CMS improved significantly after ESWT in the NCTS (78.7 ± 18.3,* p* = 0.001), TCTS (86.9 ± 19.7,* p* < 0.001), and DCTS groups (71.1 ± 17.8,* p* = 0.001) (Table 2).

**Figure 1:**
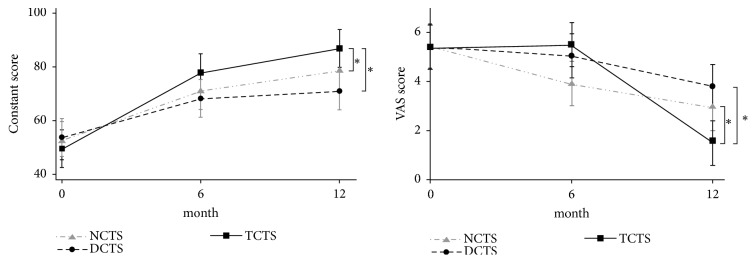
CMS and VAS 6 and 12 months after ESWT. CMS: Constant-Murley score. VAS: visual analog scale. *∗ p<0.05*.

In a comparison of functional outcome between groups, significant differences were found with regard to pain reduction and functional improvement 12 months after ESWT (Figure 1). The TCTS group (from 5.4 ± 1.04 to 1.5 ± 2.48) presented a greater reduction in the VAS than the NCTS (from 5.5 ± 0.76 to 2.9 ± 2.86) and DCTS groups (from 5.4 ± 0.94 to 3.8 ± 2.46) (p = 0.011). In terms of functional outcome, the overall CMS was superior in the TCTS group as compared with the NCTS and DCTS groups (86.9 ± 19.7 versus 78.7 ± 18.3 and 71.1 ± 17.8) (*p* = 0.014). In addition, significantly greater improvements were observed for the subscales of pain (8.8 ± 2.00 versus 7.7 ± 2.13 and 6.5 ± 2.16,* p* = 0.008), strength (22.4 ± 5.10 versus 19.2 ± 5.22 and 18.4 ± 4.69,* p* = 0.004), and range of motion (33.9 ± 7.77 versus 31.7 ± 7.18 and 28.3 ± 7.06,* p* = 0.030) in the TCTS group as compared with the NCTS and DCTS groups. However, the scores of the subscales of night pain (4.3 ± 1.13 and 4.2 ± 1.04 versus 3.3 ± 1.20,* p *= 0.012) and activity (17.6 ± 4.24 and 16.1 ± 4.16 versus 14.7 ± 4.13,* p* = 0.031) were similar in the TCTS and NCTS groups and superior to the DCTS group (Table 2).

With regard to overall satisfaction, 70% of the patients were complaint-free in the TCTS group, which was significantly higher than the percentages for the NCTS group (15%) and DCTS group (25%) (*p* < 0.05) (Table 3).

## 4. Discussion

The principal finding of the present study was a positive efficacy of ESWT for the treatment of chronic shoulder tendinosis, with superior outcomes for type II and type III calcification as compared with type I calcification and noncalcified tendinosis. ESWT is a popular advanced conservative treatment for chronic tendinosis with promising results, especially for the treatment of calcified tendinosis of the shoulder [16, 17]. However, several studies have failed to demonstrate significant outcomes of ESWT for the treatment of noncalcified tendinosis of the shoulder, and nor has a difference in the efficacy of ESWT been identified between treatments for calcified and noncalcified tendinitis of the shoulder [15, 22].

In the present study, we conducted a match-controlled group comparison of the effect of high-energy ESWT in NCTS, DCTS, and TCTS cases. The improvement was significant in each group after ESWT with regard to shoulder pain and function. These findings were in accordance with current literature regarding treatment for calcified tendinosis of the shoulder. Gerdsmeyer et al. [23] reported beneficial effects with respect to pain, shoulder function, and calcium resorption of both low- and high-energy ESWT. In a meta-analysis conducted by Verstraelen et al. [24], high-energy ESWT was found to result in greater improvement of the Constant score and higher radiographic resorption in comparison with low-energy ESWT. Regarding NCTS, our study revealed significant improvements in the Constant score and the pain score after ESWT, which were in line with the findings of previous studies. Galasso et al. [25] and Kolk et al. [26] reported significant improvements for noncalcified shoulder tendinosis in both the pain score and Constant score at 3 and 6 months after ESWT. Nevertheless, both studies illustrated that the results did not differ from those of a placebo group.

Comparing the constant score and pain score among the groups, the TCTS group demonstrated significant pain reduction and Constant score improvement 12 months after ESWT as compared with the other two groups. In terms of calcific shoulder tendinitis, Rompe et al. [27] reported better resorption of calcium deposits in patients with type II calcification, and inferior outcomes were significant in patients with homogenous deposits in comparison with surgical extirpation. Regarding the energy level of ESWT, Peters et al. [28] demonstrated a greater effect of high-energy treatment, with a higher resorption rate of calcium deposits; they also reported pain recurrence in 87% of patients in the low-energy group with residual calcium deposits, whereas no residual deposits or recurrent pain were observed in the high-energy group. In our study, the calcification was more homogenous and dense in the DCTS group, whereas in the TCTS group, it was inhomogeneous, and of a translucent and cloudy appearance; and superior outcomes were observed in the TCTS group, which was in line with existing literature.

**Figure 2:**
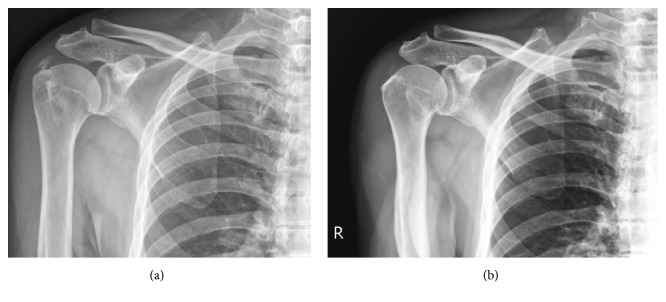
Application of ESWT on type II calcific shoulder tendinosis. (a) Plain film before ESWT (b) 6 months after ESWT. Complete resorption of calcium deposits with improvement of CMS from 60 to 88 and VAS from 5 to 1.

**Figure 3:**
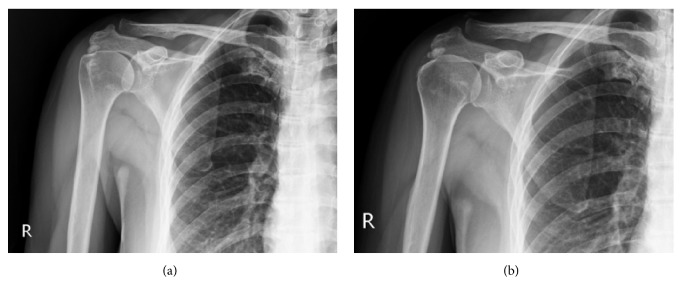
Application of ESWT on type I calcific shoulder tendinosis. (a) Plain film before ESWT (b) 6 months after ESWT. No resorption of calcium deposits without interval change regarding CMS and VAS.

Though several tentative theories have been proposed, the exact mechanism of ESWT in tendinosis remains uncertain. Some studies have shown that ESWT produces a tensile force, leading to physical effects as a result of cavitation [29]. In the application of the cavitation bubble effect on calcium deposits, the integrity of the deposits might be destroyed, and they may become resorptive. The dissolution of calcification appears to be similar to the effects of subacromial decompression by acromioplasty. Additional studies demonstrated a beneficial effect of acromioplasty without resection of calcification. Tillander et al. [30] and Schiepers et al. [31] reported that calcification disappeared after isolated acromioplasty. Although the need for additional acromioplasty after calcium resection is still under debate, several studies have demonstrated a trend of better pain reduction after acromioplasty in patients with mechanical subacromial irritation [32, 33]. In clinical studies, the application of ESWT for calcific tendinitis resulted in superior outcomes in patients with type II calcification as compared with type I calcification, with a higher resorption rate in patients with inhomogeneous calcium deposits [27]. Therefore, the decompressive effect might be more prominent in type II or type III calcification owing to the greater resorption rate, leading to improvements in outcome after ESWT. Regarding noncalcific shoulder tendinosis, we cannot expect a decompressive effect, but the effect of neovascularization might contribute to functional and pain improvement after ESWT. In this study, the TCTS group had a greater Constant score improvement and higher pain reduction, with inferior outcomes in the DCTS and NCTS groups. In line with the existing literature, a lesser mechanical decompressive effect was expected in the DCTS and NCTS groups, which might have contributed to the inferior outcomes. The representative cases demonstrated complete resorption of calcium deposits in type II calcified shoulder tendinosis patients with great improvement of outcomes and no resorption of calcification in type I calcified shoulder tendinosis without clinical improvement 6 months after high-energy ESWT (Figures 2 and 3).

There were some limitations in this study. First, although the case numbers in the present study were sufficient to demonstrate statistical differences in functional outcome, the limited numbers might weaken the power of the results. Second, though we observed significant improvement in each group after ESWT, the nature of spontaneous regression in calcific and noncalcific shoulder tendinitis might have the potential to cause bias in the study when no sham group was included. Third, advanced radiologic follow-up, such as sonography or MRI, was not performed in every case owing to the retrospective study design. However, we believe that the clinical functional outcome is still the major concern, rather than the radiographic outcome, for attending clinical physicians.

## 5. Conclusion

The present match-controlled analysis demonstrated a positive efficacy of ESWT for chronic shoulder tendinosis, with superior outcomes in patients with type II and type III calcification as compared with type I calcification and noncalcified tendinosis at the 1-year follow-up point. The results indicated that an alternative procedure to ESWT should be considered for patients with type I calcification and noncalcified tendinitis owing to the lower satisfaction rate following treatment in these patients.

## Figures and Tables

**Table 1:** Demographic data.

	NCTS	TCTS	DCTS	*p* value
	*n* = 20	*n* = 20	*n*= 20	
Age, years (mean ± SD, range)	52.4 ± 7.24(42-68)	53.3 ± 93.58 (32-78)	53.0 ± 10.98 (32-78)	0.971
Gender (M/F)	7/13	7/13	6/14	1.000
Side of lesion (right/left)	11-9	12/8	12/8	1.000
Duration of symptoms, months (mean ± SD, range)	13.4 ± 12.65 (6-56)	9.8 ± 9.3 (6-48)	20.3 ± 20.90 (6-96)*∗*	0.016

NCTS: noncalcified tendonosis of shoulder.

TCTS: calcified tendinosis types II and III of shoulder.

DCTS: calcified tendinosis type I of shoulder.

*∗*p < 0.05 versus TCTS.

**Table 2:** Pain score and functional outcomes one year after ESWT.

Clinical assessment	NCTS	TCTS	DCTS	*P value*
	*n*=20	*n* =20	*n* =20	
*VAS*				
Before Tx (SD; range)	5.5 ± 0.76(4-7)	5.4 ± 1.04(2-8)	5.4 ± 0.94(3-7)	.944
After Tx (SD; range)	2.9 ± 2.86(0-9)*∗*	1.5 ± 2.48(0-6)	3.8 ± 2.46(0-6)*∗*	.011
P value	.001	<.001	.020	
*Pain score*				
Before Tx (SD; range)	4.6 ± 0.88(2-6)	4.1 ± 1.00(2-6)	4.6 ± 0.60(3-5)	.091
After Tx (SD; range)	7.7 ± 2.13(3-10)*∗*	8.8 ± 2.00(4-10)	6.5 ± 2.16(4-10)*∗*	.004
P value	.001	<.001	.003	
*Night pain*				
Before Tx (SD; range)	2.6 ± 0.83(1-4)	2.6 ± 0.94(1-4)	2.6 ± 0.50(2-3)	.985
After Tx (SD; range)	4.2 ± 1.04(2-5) §	4.3 ± 1.13(2-5)	3.3 ± 1.20(2-5)*∗*	.012
*Strength*	<.001	<.001	.030	
Before Tx (SD; range)	12.6 ± 3.66(5-20)	11.2 ± 3.23(5-16)	12.7 ± 3.08(7-18)	.349
After Tx (SD; range)	19.2 ± 5.22(8-25)*∗*	22.4 ± 5.10(8-25)	18.4 ± 4.69(10-25)*∗*	.002
P value	<.001	<.001	.001	
*Activity*				
Before Tx (SD; range)	10.8 ± 3.16(5-5)	10.0 ± 2.51(6-16)	10.7 ± 2.56(6-16)	.626
After Tx (SD; range)	16.1 ± 4.16(10-23) §	17.6 ± 4.24(6-20)	14.7 ± 4.13(8-20)*∗*	.031
P value	.001	<.001	.002	
*Motion*				
Before Tx (SD; range)	22.0 ± 8.41(8-34)	21.9 ± 7.99(6-36)	23.3 ± 5.20(16-36)	.721
After Tx (SD; range)	31.7 ± 7.18(14-38)*∗*	33.9 ± 7.77(16-40)	28.3 ± 7.06(18-40)*∗*	.030
P value	.002	<.001	.005	
*Constant*				
Before Tx (SD; range)	52.5 ± 14.5(21-74)	49.7 ± 9.03(33-62)	53.8 ± 7.66(42-64)	.409
After Tx (SD; range)	78.7 ± 18.3(38-98)*∗*	86.9 ± 19.7(40-100)	71.1 ± 17.8(44-98)*∗*	.007
P value	.001	<.001	.001	

*∗p < 0.05* versus TCTS (calcified tendinitis types II and III).

§  *p < 0.05* versus DCTS(calcified tendinitis type I).

NCTS: noncalcified tendonosis of shoulder.

TCTS: calcified tendinosis type II and III of shoulder.

DCTS: calcified tendinosis type I of shoulder.

**Table 3:** Overall satisfaction rate one year after ESWT.

	NCTS	TCTS	DCTS	*P value*
	*n* (%)	*n* (%)	*n* (%)	
Complaint-free	3(15)	14(70)*∗*	5(25)	0.006
Significantly better	7(35)	1(5)	6(30)	
Slightly better	5(25)	1(5)	2(10)	
Unchanged	5(25)	4(20)	7(35)	

*∗*The adjusted standardized residual was greater than 2, *p <0.05.*

NCTS: noncalcified tendonosis of shoulder.

TCTS: calcified tendinosis type II and III of shoulder.

DCTS: calcified tendinosis type I of shoulder.

## Data Availability

The data used to support the findings of this study are available from the corresponding author upon request.
